# Mitochondrial Respiration in *Drosophila* Ovaries after a Full Cycle of Oogenesis under Simulated Microgravity

**DOI:** 10.3390/cimb43010015

**Published:** 2021-05-22

**Authors:** Irina V. Ogneva, Maria A. Usik

**Affiliations:** 1 Cell Biophysics Laboratory, State Scientific Center of the Russian Federation Institute of Biomedical Problems of the Russian Academy of Sciences, 76a, Khoroshevskoyoe Shosse, 123007 Moscow, Russia; usik.maria@mail.ru; 2 Medical and Biological Physics Department, I. M. Sechenov First Moscow State Medical University, 8-2 Trubetskaya St., 119991 Moscow, Russia

**Keywords:** ovary, cell respiration, cytoskeleton, simulated microgravity, fruit fly

## Abstract

Studies of the function of the female reproductive system in zero gravity are urgent for the future exploration of deep space. Female reproductive cells, oocytes, are rich in mitochondria, which allow oocytes to produce embryos. The rate of cellular respiration was determined to assess the functional state of the mitochondrial apparatus in *Drosophila melanogaster* ovaries in which the full cycle of oogenesis took place under simulated microgravity. Since cellular respiration depends on the state of the cytoskeleton, the contents of the main cytoskeletal proteins were determined by Western blotting. To modulate the structure of the cytoskeleton, essential phospholipids were administered per os at a dosage of 500 mg/kg in medium. The results of this study show that after a full cycle of oogenesis under simulated microgravity, the rate of cellular respiration in the fruit fly ovaries increases, apparently due to complex II of the respiratory chain. At the same time, we did not find any changes in the area of oocytes or in the content of proteins in the respiratory chain. However, changes were found in the relative contents of proteins of the actin cytoskeleton. There were no changes of essential phospholipids and no increase in the rate of cellular respiration of the ovaries after exposure to simulated microgravity. However, in the control, the administration of essential phospholipids led to a decrease in the efficiency of oxygen consumption in the flies’ ovaries due to complexes IV–V.

## 1. Introduction

Realization of reproductive function after, or even during, a long space flight is one of the prerequisites for maintaining the human species during the exploration of deep space. Among a number of negative factors of space flight beyond leaving the Earth’s magnetosphere, ionizing radiation, hypomagnetic conditions and weightlessness are included. Moreover, weightlessness deserves special attention due to the technically extreme difficulty in providing protection against it.

Under space flight conditions in a low-earth orbit (LEO), where microgravity is the main factor and in experiments simulating the conditions of weightlessness on Earth, it has been convincingly shown that a number of changes can be observed at the level of germ cells and organs. Thus, in space flight, the number of mature spermatozoa in the epididymis of the testes decreases and in model experiments, for example, with antiorthostatic suspension, this decrease is more significant [1,2,3,4,5,6]. Changes in the contents of various cytoskeletal proteins and the expression of the corresponding genes have been observed [7,8].

The female reproductive system is even more vulnerable to various negative factors due to the limited supply of primary germ cells, particularly in humans. However, in general, female reproductive cells and organs under zero gravity have not been studied. Using the antiorthostatic suspension model to simulate the effects of weightlessness on female mice, we showed that regulation of cytoskeletal organization occurs primarily at the translation level, maintaining the protein pattern unchanged but with a predominant increase in the mRNA contents of the corresponding genes [9]. In oocytes, blebbing and delayed maturation are observed [10] as are vacuolated mitochondria [11]. At the same time, oocytes provide mitochondria and the future energy supply to embryos; during oogenesis, mitochondria are selected and the selected variants are intensively increased [12]. Nevertheless, practically nothing is known about the state of the mitochondrial apparatus in oocytes and ovaries under zero gravity, which may be associated with a number of methodological difficulties when working with mammalian oocytes.

A convenient model for this kind of research is the fruit fly *Drosophila melanogaster*, which can be exposed to simulated microgravity conditions during the full cycle of oogenesis. Despite a number of differences in oogenesis compared to that in mammals, the main features of maintaining the energy supply of oocytes and early embryos are apparently rather conserved between these species. Mitochondrial DNA begins to replicate after the initiation of oocyte maturation in the ovary and, in particular in *Drosophila melanogaster*, is controlled by the JNK-insulin-Myc cascade [13]. Therefore, the study of the functional state of the mitochondrial apparatus in the ovaries after a full cycle of oogenesis under conditions simulating the effects of microgravity can be of key importance.

At the same time, the functional activity of the mitochondrial apparatus depends on a number of factors, including the state of the cytoskeleton [14]. Under conditions of real and simulated microgravity, microfilaments and microtubules are disorganized in various types of cells [15,16,17,18], intermediate filaments are altered and the mitochondrial localization is disturbed [19]. In experiments with muscle cells and male reproductive cells, it has been shown that microgravity conditions lead to various changes in cellular respiration [20,21,22,23]. However, the administration of essential phospholipids can prevent both changes in the structure of the cytoskeleton and cellular respiration [7,24,25].

In connection with the above findings, the main goal of this study was to determine the cellular respiration and contents of the main cytoskeletal proteins in the ovaries of flies that had undergone a full cycle of oogenesis under conditions of microgravity simulation and after of oral administration of essential phospholipids.

## 2. Materials and Methods

### 2.1. Experimental Design

To study the full cycle of oogenesis, virgin females of the adult fruit fly *Drosophila melanogaster* of the Canton S line, 2 days old, were placed in 50 mL Falcon tubes (40 individuals in each tube) with an air-permeable lid containing 10 mL of the standard medium for *Drosophila* breeding. Essential phospholipids were added to half of the tubes in the form of EssentsialeR ForteN (A. NATTERMANN and Cie. GmbH, Koln, Germany) at a dosage of 500 mg/kg medium [25]. The introduction of essential phospholipids began at least 3 generations before the experiment was started in such a way that all the stages of germ cell development took place in modified nutrient medium. Four study groups were formed:

- C: Control group receiving the standard nutrient medium and kept under standard conditions;
- sµg: A group that received the standard nutrient medium and remained in simulated microgravity for a full cycle of oogenesis;
- C + E: Control group receiving nutrient medium supplemented with essential phospholipids and kept under standard conditions;
- sµg + E: A group receiving a nutrient medium supplemented with essential phospholipids that was exposed to simulated microgravity for a full oogenesis cycle.

The effects of zero gravity were reproduced by exposing flies on a random positioning machine (Gravite^®^, GC-US-RCE010001, Space Bio-Laboratories Co., Ltd., Hiroshima, Japan). It has been shown that the effects of such an exposure correlate with the data obtained after a real space flight [26]. A mode was used that ensured that the superposition of the orientation of the tubes with flies relative to the gravity vector is zero, on average, for 15 s. The fly tubes were placed in the center of the platform and exposed under the same conditions as the control groups.

At the end of the full cycle of oogenesis, the ovaries were extirpated. For some of the ovaries (at least 20 from each replicate, with at least 7 biological replicates), an experimental procedure was immediately performed to determine the rate of cellular respiration. Other ovaries (at least 10 from each replicate, at least 7 biological replicates) were frozen in liquid nitrogen and then used for protein isolation and subsequent Western blotting. In addition, some of the native ovaries were used to estimate the area of oocytes.

All the experimental procedures were approved by the Commission on Biomedical Ethics of the State Scientific Center of the Russian Federation—Institute of Biomedical Problems (IBMP) (Minutes No. 521 dated 25 September 2019).

### 2.2. Estimation of Cellular Respiration by Polarography

To analyze cellular respiration, the oxygen uptake rate was determined by polarography according to the protocol described in detail by Kuznetsov A.V. et al. [27] with minor modifications for the genital tissues of *Drosophila melanogaster* [25]. Each sample contained at least 20 ovaries; at least 7 biological replicates were performed for each experimental group.

Briefly, the ovaries were isolated in physiological solution and saponin was added at a final concentration of 10 μg/mL, incubated for 15 min at +22 °C and transferred to a polarographic cuvette to measure the change in oxygen concentration using Oxygraph+ (Hansatech Instruments Ltd., King’s Lynn, Norfolk, UK).

We recorded V0—the rate of oxygen uptake by permeabilized ovaries and Vglu + mal—the rate of oxygen uptake upon addition of the substrates of the first complex of the respiratory chain 10 mM glutamate and 5 mM malate; Vmax is the maximum oxygen uptake rate (when adding 2 mM ADP).

Then, a substrate-inhibitor analysis was performed [27], adding inhibitors of complexes of the respiratory chain (0.5 μM rotenone, an inhibitor of complex I; 5 μM antimycin, an inhibitor of complex III) and substrates of subsequent complexes (10 mM succinate, a substrate of complex II; 0.5 mM TMPD + 2 mM ascorbate—artificial substrates of complex IV) and recording the rate of oxygen uptake by V(II) and V(IV). After the substrate-inhibitory analysis, each sample was tested for the intactness of the outer mitochondrial membrane by adding 10 μM cytochrome *c*. The cellular respiration rate is expressed as pmol O_2_ per mL per min per ovary.

### 2.3. Estimation of the Oocyte Area

Immediately after extirpation, the ovaries were placed on a glass slide and photographed under transmitted light under an Olympus IX73 inverted microscope (Olympus Corporation, Tokyo, Japan) using 10× magnification. The following stages were determined: germarium—G, S2, S3, S4, S5, S6, S7, S8, S9, S10, S11, S12, S13 and S14, in accordance with [28]. The area was counted in µm^2^ using ImageJ open source software (version for Windows, https://imagej.net/Fiji, accessed on 30 April 2021). Due to the significant increase in the area of oocytes from stage S2 to S14, the data are presented on a logarithmic scale (lnS).

### 2.4. Evaluation of the Protein Content by Western Blotting

Frozen ovarian tissues were homogenized in Laemmli buffer containing a protease inhibitor cocktail (Calbiochem, San Diego, CA, USA). After electrophoresis in a SDS-polyacrylamide gel, proteins were transferred onto nitrocellulose membranes, followed by staining with specific primary antibodies:

- Mouse antibodies against cytochrome *c*-1, MW 13.5 kDa (at a concentration of 5 μg/mL, Abcam, Cambridge, UK, #ab13575); cytochrome *c* oxidase, MW 16 kDa (at a concentration of 1 μg/mL, Abcam, Cambridge, UK, #ab14744); and ATPsyntase F1 (blw), MW 56 kDa (at a concentration of 1 μg/mL, Abcam, Cambridge, UK, #ab14748);
- Rabbit antibodies against alphaTub84, MW 50 kDa (diluted 1:1000, Abcam, Cambridge, UK, #ab52866); betaTub56D, MW 50 kDa (diluted 1:1000, Abcam, Cambridge, UK, #ab179513); and beta-actin, MW 42 kDa (diluted 1:10,000, Abcam, Cambridge, UK, #ab227387);
- rat antibodies against alpha-actinin MW 102 kDa (at a concentration of 1 μg/mL, Abcam, Cambridge, UK, #ab50599).

Mouse secondary antibodies against, rabbit primary antibodies (#7076 and #7074, respectively, Cell Signaling Technology, Danvers, MA, USA) and HRP-conjugated streptavidin-peroxidase were used. The membranes were developed using ECL substrate (Bio-Rad Laboratories, Inc., Hercules, CA, USA). Signals were detected using a ChemiDoc XRS+ imaging system (Bio-Rad Laboratories, Inc., Hercules, CA, USA) and analyzed using Image Lab Software (Bio-Rad Laboratories, Inc., Hercules, CA, USA).

Biotinylated goat antibodies were used as the secondary antibodies to detect rat IgG ((Sigma, Merck, Darmstadt, Germany, #B7139) at a dilution of 1:10,000. The membranes were then treated with a streptavidin solution conjugated with horseradish peroxidase (Sigma, Merck, Darmstadt, Germany, #E2886) at a dilution of 1:10,000. The protein bands were revealed using 3,3′-diaminobenzidine (Amresco Inc., Solon, OH, USA, #E733-50) and the data were analyzed using the ImageJ program (version for Windows, https://imagej.net/Fiji, accessed on 30 April 2021).

### 2.5. Statistical Analysis

The results were statistically analyzed by ANOVA using the post hoc t-test with a significance level of *p* < 0.05 to assess the reliability of differences between groups. The data are presented as the mean ± standard error of the mean (M ± SE).

All the methods were carried out in accordance with the relevant guidelines and regulations.

## 3. Results

### 3.1. Cellular Respiration Rate and Oocyte Area

**Figure 1:**
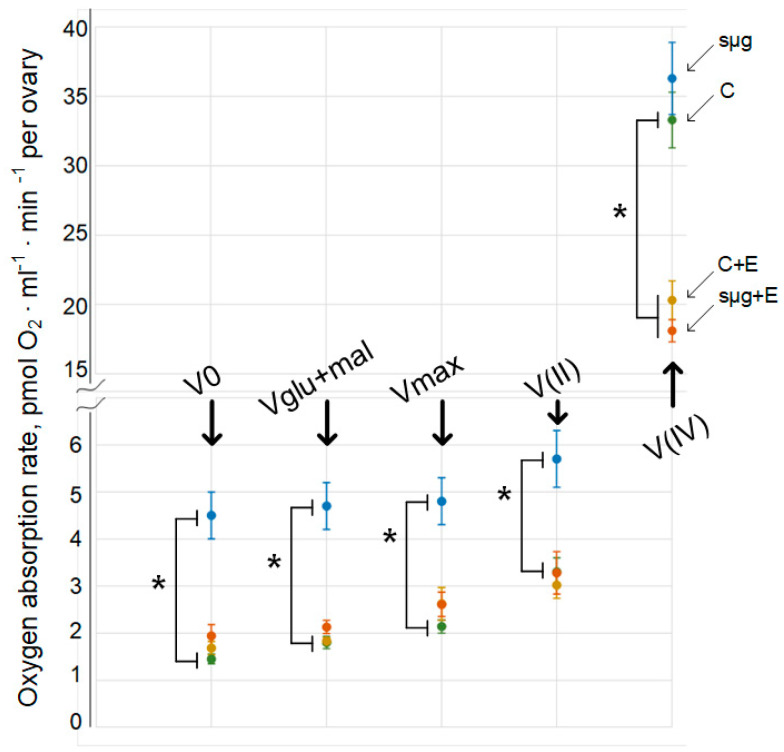
Respiration rate in ovaries after exposure to simulated microgravity conditions during the full cycle of oogenesis. C—control group, green line; sμg—fly group that had undergone the full cycle of oogenesis under simulated microgravity, blue line; C + E—control group that received essential lipids per os (500 mg per 1 kg medium), yellow line; sμg + E—fly group that received essential phospholipids and had undergone the full cycle of oogenesis under simulated microgravity, red line. V0—respiration rate of permeabilized cells; Vglu + mal—respiration rate after adding 10 mM glutamate + 5 mM malate; Vmax—maximum respiration rate after adding 2 mM ADP; V(II)—respiration rate after adding 0.5 μM rotenone (complex I inhibitor) and subsequent supplementation with 10 mM succinate (substrate of complex II); V(IV)—respiration rate after adding 5 μM antimycin (complex III inhibitor) and subsequent supplementation with 0.5 mM TMPD + 2 mM ascorbate (artificial substrates of complex IV). * *p* < 0.05 in comparison with the control group.

The cellular respiration rate of permeabilized *Drosophila melanogaster* ovaries (V0) that had undergone a full cycle of oogenesis under simulated microgravity (sμg group) was 210% higher than that of ovaries in the control group (*p* < 0.05) (Figure 1). Similarly, the cellular respiration rate was increased by 161% with the addition of 10 mM glutamate + 5 mM malate (Vglu + mal) (*p* < 0.05) and the maximum respiration rate was increased by 161% with the addition of 2 mM ADP (Vmax) (*p* < 0.05). Substrate-inhibitory analysis showed that after inhibition of the first complex of the respiratory chain with 0.5 μM rotenone and the subsequent addition of the substrate of complex II (10 mM succinate), the rate of cellular respiration V(II) was also increased by 72% compared to that of the control group (*p* < 0.05). However, after inhibition of complex III with 5 μM antimycin and the addition of artificial substrates of complex IV (0.5 mM TMPD + 2 mM ascorbate), the rate of oxygen uptake V(IV) in the sμg group did not differ from that in the control group.

In the groups that were administered essential lipids, both the control group (group C + E) and the group that had been exposed to simulated microgravity during the full cycle of oogenesis (group sμg + E), the oxygen uptake rates V0, Vglu + mal and Vmax did not differ from those in group C (Figure 1). Moreover, substrate-inhibitor analysis showed that V(II) in groups C + E and sμg + E also did not differ from that in control group C. However, V(IV) did not differ between groups C + E and sμg + E but decreased relative to that in control group C by 39% and 46% (*p* < 0.05), respectively.

**Figure 2:**
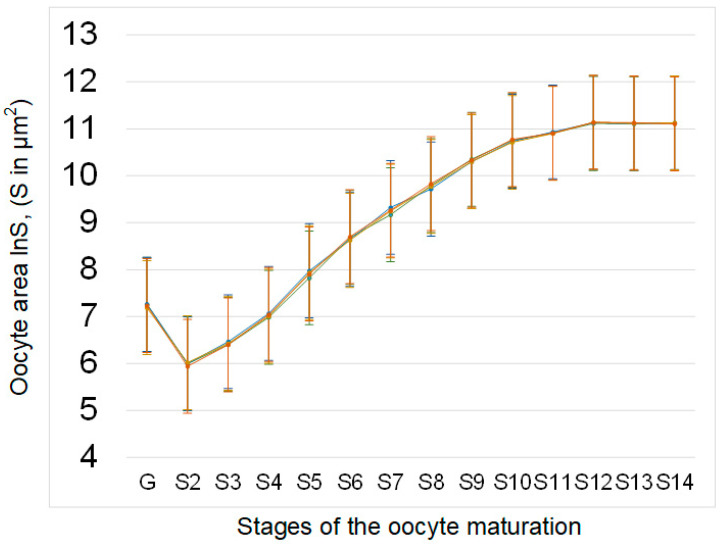
Area of oocytes at different stages of development in the ovaries of *Drosophila melanogaster*. The oocyte areas (measured in μm^2^; standard error for each value does not exceed 10%) are plotted on a logarithmic scale (lnS) due to the significant increase in values from early stages to mature oocytes. The color coding of the groups is the same as above (C—green, sμg—blue; C + E—yellow; sμg + E—red). There were no significant differences in the square of oocytes at different stages between the experimental groups.

The area of oocytes at different stages of development (from germarium G to mature oocyte S14) in the sμg group and the groups administered essential phospholipids (C + E and sμg + E) did not significantly differ from that in control group C (Figure 2).

### 3.2. The Relative Contents of Proteins Involved in Cellular Respiration and Cytoskeleton Organization

**Figure 3:**
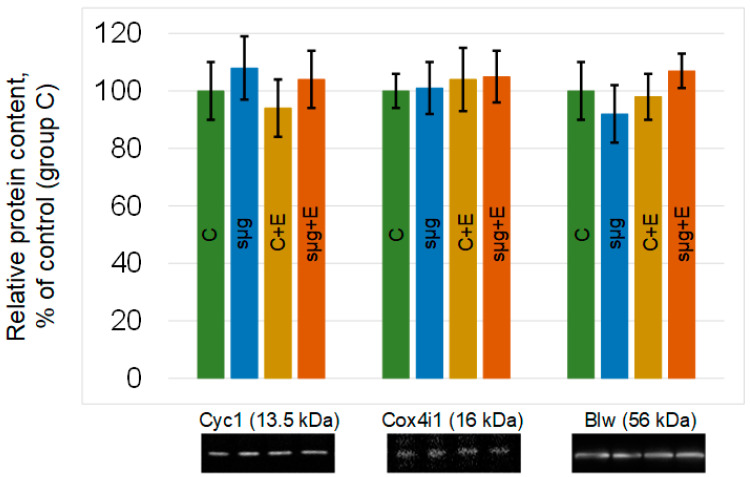
Relative contents of proteins participating in mitochondrial respiration. Cytochrome *c*-1 (CG4769), 13.5 kDa, is located between complexes III and IV; cytochrome *c* oxidase (CG10396), 16 kDa, is the protein of complex IV; and blw (bellwether), 56 kDa, is the subunit of ATP synthase. The color coding of the groups is the same as above (C—green, sμg—blue; C + E—yellow; sμg + E—red). Typical Western blots for each protein are shown under the corresponding part of the histogram.

The relative content of proteins that are components of the III–V complexes of the respiratory chain did not change in the ovaries of *Drosophila melanogaster* that had undergone a full cycle of oogenesis under simulated microgravity. The administration of essential phospholipids also did not lead to significant changes in the relative content of cytochrome *c*-1, cytochrome *c* oxidase, or ATP synthase F1 in groups C + E and sμg + E compared to those in group C (Figure 3).

**Figure 4:**
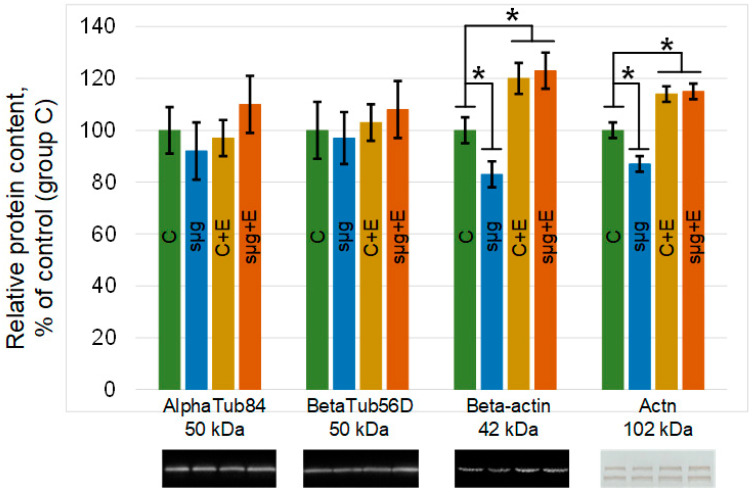
Relative contents of cytoskeletal proteins in *Drosophila melanogaster* ovaries. AlphaTub84, alpha-tubulin84D and alpha-tubulin84B, 50 kDa, are components of the tubulin heterodimer; betaTub56D, beta-tubulin, 50 kDa, is a component of the tubulin heterodimer; Beta-actin, Act57B and Act87E, 42 kDa, are components of the microfilament network; Actn, alpha-actinin, 102 kDa, is a component of the microfilament network. The color coding of the groups is the same as above (C—green, sμg—blue; C + E—yellow; sμg + E—red). * *p* < 0.05 in comparison with the control group C. Typical Western blots for each protein are shown under the according part of the histogram.

Likewise, there was no change in any of the study groups of the major components of microtubules: alpha- and beta-tubulin (Figure 4). However, the contents of the components of the microfilament network changed. The relative contents of beta-actin and alpha-actinin in the ovaries of flies that had undergone a full cycle of oogenesis under simulated microgravity decreased by 17% and 13% (*p* < 0.05), respectively, compared to the control (group sμg vs. group C). The administration of essential phospholipids led to an increase in the contents of actin and alpha-actinin in the control group C + E compared with those in group C by 20% and 14% (*p* < 0.05), respectively. However, when exposed to simulated microgravity and administered essential phospholipids, the relative contents of these proteins did not change compared to those in the corresponding control group (group sμg + E vs. group C + E), remaining 23% higher than the content in group C (beta-actin, *p* < 0.05) and 15% (alpha-actinin, *p* < 0.05).

## 4. Discussion

The study of the influence of space flight factors on the functional state of the ovaries and oocytes can provide fundamentally new information about the role of gravity in maintaining the reproductive potential of the human species. Since mitochondria are key participants in metabolism and provide the cell with energy, we assessed cellular respiration as an indicator of the functional state of *Drosophila melanogaster* ovaries that had undergone a full cycle of oogenesis under conditions simulating the effects of microgravity.

The results obtained indicate that under simulated microgravity, cellular respiration of the ovaries increases (Figure 1). Since the rate of oxygen uptake was normalized to that in the ovary, the area of the ovaries was estimated: there were no differences between the control and experimental groups (Figure 2). In addition, there were no differences between the contents of respiratory chain proteins, such as cytochrome *c* (complex III), cytochrome *c* oxidase (complex IV) and blw (ATPsynatse subunit) (Figure 3). Therefore, an inhibitory analysis was carried out to determine in which part of the respiratory chain the increase in the efficiency of cellular respiration occurs.

Since the use of an inhibitor of complex I of the respiratory chain and the subsequent addition of substrates of complex II also leads to an increased rate of oxygen uptake, but V(IV) does not differ from that in the control, it can be assumed that the total increase occurs, apparently, due to complex II of the respiratory chain. Complex II of the respiratory chain, in contrast to proteins of other complexes, is completely encoded by nuclear DNA and is one of the key sensors of apoptosis induction [29]. At the same time, it should be noted that apoptosis in the ovaries of *Drosophila melanogaster* is observed at three stages of development and is a necessary stage in the normal maturation of oocytes [30]; therefore, disturbances in its induction may be critical for the normal maturation of oocytes.

A number of studies have shown that the transcription factor STAT3 can increase the efficiency of respiration as a result of its action on complex I and/or complex II, both in differentiated cells [31,32] and in pluripotent cells [33]. However, in this case, it remains unclear how STAT3, which is localized in the cytoplasm, is activated.

Previously, it was assumed that the destruction of actin could activate STAT3 [34]. However, the same authors refined their data and showed that actin-associated alpha-actinin more significantly activated STAT3 in *Drosophila* [35]. Moreover, in mammals, alpha-actinin3, a homolog of *Drosophila* alpha-actinin, also colocalizes with STAT3 in the heart [36]. Therefore, in this work, we analyzed the contents of a number of cytoskeletal proteins, primarily alpha-actinin.

**Figure 5:**
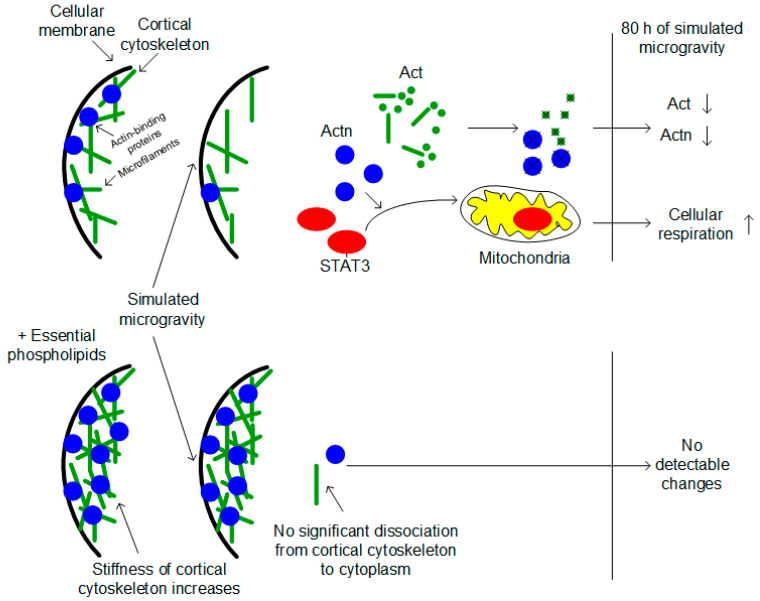
Hypothesis of the possible mechanism of increasing cellular respiration of *Drosophila* ovaries after full cycle of oogenesis under simulated microgravity. Changes of the external mechanical load leads to cortical cytoskeleton deformation and migration actin-binding proteins and actin filaments to the cytoplasm. Actin-binding proteins, particularly alpha-actinin Actn, recruits factor STAT3. It migrates to the mitochondria and activates cellular respiration. The administration of the essential phospholipids increases stiffness of the cortical cytoskeleton and prevents deformation under changes of the mechanical load.

The tubulin content did not change; however, the total actin and alpha-actinin contents were significantly reduced in the ovaries after simulating the effects of microgravity (Figure 4). Since STAT3 is activated by alpha-actinin after 6 h [35], it can be assumed that long-term exposure during the full cycle of oogenesis demonstrates an adaptive pattern. Earlier, in a number of works, it was assumed that one of the first events of mechanotransduction was the dissociation of alpha-actinin from cortical actin and its increase in the cytoplasm [37,38]. This transient accumulation could lead to the activation of STAT3 and an increase in the rate of cellular respiration (Figure 5). Moreover, we observed the dissociation of alpha-actinin-1 and an increase in the rate of cellular respiration in the heart muscle when simulating microgravity in rats [22,39]. Furthermore, the content of alpha-actinin decreased with the decreasing content of actin, which we also observed in the present study.

To test this hypothesis in vivo, oral administration of essential lipids was used to alter the structure of the cortical cytoskeleton. The use of essential phospholipids leads to a decrease in the content of cholesterol in the membrane and, further, to rearrangements of the cortical cytoskeleton, causing an increase in its stiffness and in the contents of actin and actin-binding proteins [24]. In particular, by introducing essential phospholipids, we managed to prevent the dissociation of alpha-actinin from the cortical cytoskeleton of soleus muscles in rats [24], as well as in mice and *Drosophila melanogaster*, leading to changes in the structure of spermatozoa and spermogram parameters when simulating microgravity [7,25]. In this study, oral administration of essential phospholipids resulted in increased levels of actin and alpha-actinin in the ovaries of *Drosophila melanogaster* (Figure 4). However, no changes in the contents of these cytoskeletal proteins after exposure to simulated microgravity were observed or in changes in cellular respiration at the level of the I–IV complexes of the respiratory chain, which may support the above hypothesis regarding the role of alpha-actinin (Figure 5).

However, both in the control group and group under simulated microgravity treated with essential phospholipids, there was a decrease in the rate of ATP synthesis due to complexes IV–V of the respiratory chain according to inhibitory analysis. In the absence of changes in the area of the ovaries after administration of essential phospholipids (Figure 2), as well as in the contents of respiratory chain proteins (Figure 3), it can be assumed that the decrease in the efficiency of oxygen uptake may be associated with a change in the availability of ADP or the accumulation of ATP. ADP is transported into mitochondria by interacting with a transporter localized in the mitochondrial membrane [40,41,42]. Moreover, the carrier is associated with cardiolipin, a lipid of the inner mitochondrial membrane, a decrease in the synthesis of which reduces cellular respiration [43,44,45]. A change in the lipid composition due to the administration of essential phospholipids could lead to a decrease in the efficiency of the transporter and, accordingly, to a decrease in cellular respiration.

In summary, we have shown that after a full cycle of oogenesis under simulated microgravity in the ovaries of *Drosophila melanogaster*, cellular respiration increases in a constant area of oocytes at different stages of maturation and with constant contents of proteins of complexes III–V of the respiratory chain. The assumption about the role of the STAT3 factor in the activation of respiration due to interaction with alpha-actinin was indirectly confirmed in an experiment assessing the effects of the oral administration of essential phospholipids.

## Data Availability

All data generated or analyzed during this study are included in this article.
